# Forest understories controlled the soil organic carbon stock during the fallow period in African tropical forest: a ^13^C analysis

**DOI:** 10.1038/s41598-019-46406-2

**Published:** 2019-07-08

**Authors:** Soh Sugihara, Makoto Shibata, Antoine D. Mvondo Ze, Haruo Tanaka, Takashi Kosaki, Shinya Funakawa

**Affiliations:** 1 Graduate School, Institute of Agriculture, Tokyo University of Agriculture and Technology, Tokyo, 183-8509 Japan; 2 Graduate School of Global Environmental Studies, Kyoto University, Kyoto, 606-8501 Japan; 3 Department of Agro-Food Science, Niigata Agro-Food University, Niigata, 950-3102 Japan; 4 Faculté d’ Agronomie, Université de Dschang, B.P. 67 Dschang, Cameroon; 5 Graduate School of Global Liberal Arts, Aichi University, Aichi, 453-8777 Japan

**Keywords:** Carbon cycle, Agroecology

## Abstract

Soil organic carbon (SOC) dynamics after slash-burn agriculture are poorly understood in African tropical forest, though recent studies have revealed C4 grass invasion as a forest understory influences SOC dynamics after deforestation. This study aimed to quantify the relative SOC contribution of C4 and C3 plants separately through the sequential fallow periods of forest (cropland, or 4–7, 20–30, or >50 years of fallow forest) in the tropical forest of eastern Cameroon. We evaluated the SOC stock and natural ^13^C abundance for each layer. The SOC stock was largest in 4–7 years fallow forest (136.6 ± 8.8 Mg C ha^−1^; 100 cm depth, and C4:C3 = 58:42), and decreased with increasing fallow period. SOC from C4 plants was larger in the 4–7 and 20–30 years fallow forests (57.2–60.4 ± 5.8 Mg C ha^−1^; 100 cm depth), while it clearly decreased in >50 years fallow forest (35.0 ± 4.1 Mg C ha^−1^; 100 cm depth), resulting in the smallest SOC in this mature forest (106.4 ± 12.9 Mg C ha^−1^; 100 cm depth). These findings indicate that C4 grass understories contributed to the SOC restoration during early fallow succession in the tropical forest of eastern Cameroon.

## Introduction

The sustainability of human societies depends on the wise use of natural resources, and soil ecosystems play an important role in this management^1^. To achieve the sustainable development goals (SDGs) related to food security, climate change, and sustainable use of terrestrial ecosystems, approved by the UN General Assembly in 2015, it is critical to improve the traditional management of soil resources, based on the understanding of its importance and efficiency. In tropical Africa, the traditional slash-burn agriculture (shifting cultivation) causes substantial variation of soil organic carbon (SOC) stock, which is an important indicator not only of soil fertility but also of climate change^2–4^. In general, cultivation after the slash-burn management decreases the SOC stock and soil fertility over time, whereas fallow management, which generally requires several decades or more for reforestation, restores the SOC stock and soil fertility. For the local farmers in the African tropical forest zone, natural reforestation for long periods is likely to be a useful and single management option to restore SOC^5^. Conversion from natural forest (or woodland) to cropland has been drastically increasing under population increase during the past century throughout tropical Africa^6–8^, causing shorter fallow periods, such as from >15–60 years in the past to <15 years in the present^9^, and subsequent soil degradation^9,10^. It is therefore necessary to evaluate the SOC variations of different periods of fallow sites, based on detailed mechanisms of SOC variations in African tropical forest to allow determination of the optimal future fallow period.

Restoration of SOC during the fallow period is mainly attributed to input of litter fall by growing trees and/or understory vegetation, such as herbs and shrubs^11^. Such restoration vegetation should belong to the natural vegetation succession from herbaceous, shrubs, and pioneer tree species at the early fallow stage to climax tree species at the later fallow stage. However, the effect of understories on SOC stock variations during the above natural fallow succession period remains unclear, especially in humid Africa^12,13^. Many studies have assessed the effect of fallow management, such as from cropland to bush or forest, on SOC stock in tropical areas, and most studies observed that fallow management clearly increased the SOC, especially in the dry to sub-humid tropics^3,14^. In contrast, Kotto-same *et al*.^12^ observed that there was little SOC stock fluctuation during the various fallow stages after cultivation in humid southern Cameroon, although they found an increase of aboveground biomass in forest with a longer fallow period.

Recently, many studies have evaluated the SOC dynamics separately from invaded grass-derived C (i.e., C4-plant grass) and forest-derived C (i.e., C3-plant forest), to discuss the effect of grass vegetation on SOC dynamics in humid tropical areas^15–17^. Yonekura *et al*.^18,19^ observed that C4 grass invasion after deforestation clearly increased the SOC stock, based on the increase in grass-derived SOC, using natural ^13^C measurements in a humid tropical area of Indonesia. Navarrete *et al*.^20^ also reported that conversion from forest to pasture in Colombia increased the SOC, mainly derived from C4 grass, under low-grazing-pressure pasture. They emphasised the importance of grass management practices, which affects the amount of C input by litter, to consider the accurate SOC dynamics in the humid tropics.

Although there is little quantitative information on the effect of grass invasion on SOC restoration during fallow succession in tropical Africa, C4 grass is one of the most popular forest understories, especially in the first stage of fallow succession after slash-burn management. Moreover, C4 grasses such as *Pennisetum setaceum* and *Imperata cylindrica* (invasive species) are widely distributed in the forest–savanna transition zone of Africa and continue to extend into the deforested zone following commercial logging^21–23^. This suggests that C4 grass invasion as a primary forest understory in the first stage of fallow succession should substantially contribute to the restoration of SOC stock; however, there is little information of the time-course SOC composition through the sequential fallow period, especially in African tropical reforested areas^3,24,25^.

In this study, we hypothesised that C4 grass invasion contributes to the restoration of the SOC stock, especially in the early stage of fallow succession in humid tropical Africa. To explore the importance of C4 grass understories to SOC stock variations at each fallow stage, we investigated the SOC stock in sites with different fallow periods. Our main objective was to assess the impact of C4 grass and C3 forest residues on the SOC contents and SOC dynamics, and separately quantify the relative SOC contribution of C4 grass and C3 forest through the sequential fallow periods in the tropical forest of eastern Cameroon. In this study, we combined physical SOM fractionation and ^13^C natural abundance to quantitatively evaluate the progressive incorporation of newly deposited C (derived from C4 and/or C3 plants) into the different fractions^26–28^.

## Results

### Soil organic carbon stock in each fraction

**Figure 1:**
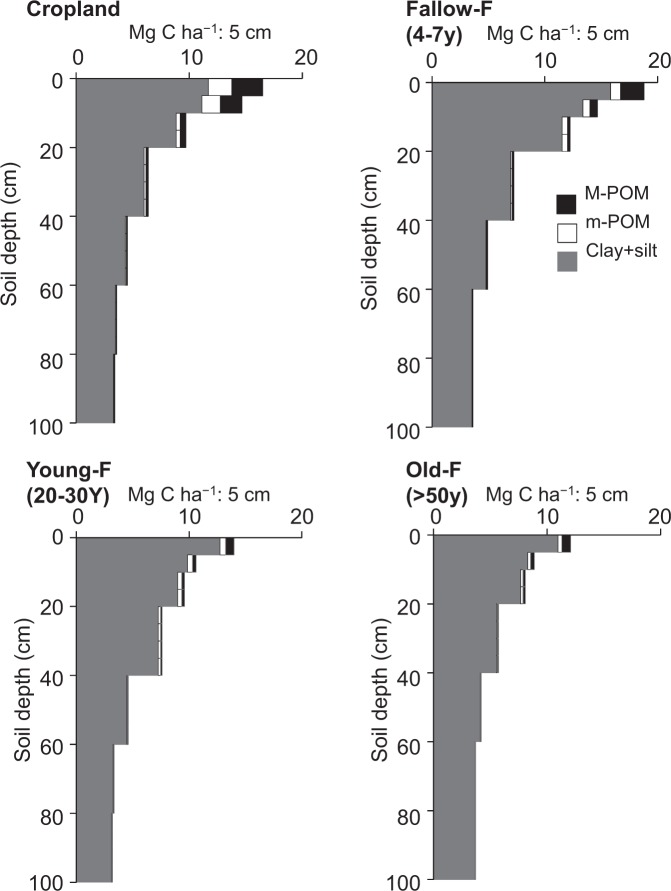
Soil organic carbon (SOC) stocks (Mg C ha^−1^ per 5 cm soil depth) in M-POM (250–2000 μm), m-POM (53–250 μm), and Clay + silt (<53 μm) fractions under cropland and different fallow period sites for the soil profile (100 cm depth) in eastern Cameroon.

Figure 1 presents the variations of SOC stock in each fraction and soil layer under the different fallow stages, and Table 1 presents the total C contents of each fraction. The SOC was mainly present in the Clay + silt fraction throughout the soil profile, and most of the SOC was observed at 0–40 cm depth. The SOC contents were largest in the surface layer (0–5 cm depth) and decreased with soil depth in all treatment plots. The SOC stock in the M-POM and m-POM in the upper layer (0–10 cm depth) was clearly larger in Cropland (4.6 ± 1.2 (standard error) and 3.7 ± 0.7 Mg C ha^−1^, respectively) than in Young-F (1.1 ± 0.2 and 0.9 ± 0.1 Mg C ha^−1^, respectively) and Old-F (1.1 ± 0.2 and 0.6 ± 0.2 Mg C ha^−1^, respectively). However, these differences were not observed in each deeper layer (<10 cm depth).

**Table 1:** Total C contents (TC) of each fraction of soils under cropland and different fallow period sites through the profiles in eastern Cameroon.

(gC kg^−1^)	Cropland				Fallow-F (4–7 y)				Young-F (20–30 y)				Old-F (>50 y)			
	M-POM	m-POM	Clay + silt	Total	M-POM	m-POM	Clay + silt	Total	M-POM	m-POM	Clay + silt	Total	M-POM	m-POM	Clay + silt	Total
0–5 cm	4.9a (1.6)	3.7 a (1.0)	21.2 b (1.3)	29.9 b (3.8)	4.2 a (0.7)	3.6 a (0.1)	32.2 a (1.5)	40.1 a (2.1)	1.5 b (0.2)	1.9 b (0.3)	26.3 ab (2.6)	28.5 b (3.3)	1.5 b (0.3)	1.2 b (0.3)	21.8 b (3.1)	24.5 b (3.3)
5–10 cm	3.2 a (1.3)	2.7 (1.0) a	18.6 ab (1.7)	24.6 a (3.7)	1.1 b (0.1)	1.0 b (0.1)	22.5 a (2.0)	24.6 a (2.0)	0.6 c (0.1)	0.8 b (0.2)	18.0 ab (2.1)	19.4 ab (2.4)	0.5 c (0.1)	0.4 c (0.1)	13.9 b (2.8)	14.9 b (2.9)
10–20 cm	0.7 (0.2)	0.5 (0.1)	12.9 b (1.4)	14.1 ab (1.6)	0.8 (0.1)	0.8 (0.2)	18.4 a (1.0)	19.9 a (1.2)	0.4 (0.1)	0.6 (0.1)	15.6 ab (1.6)	16.6 ab (1.7)	0.3 (0.01)	0.3 (0.1)	12.1 b (2.2)	12.8 b (2.3)
20–40 cm	0.3 (0.1)	0.2 (0.03)	8.2 c (0.3)	8.7 (0.4)	0.2 (0.1)	0.2 (0.03)	10.1 ab (0.6)	10.5 (0.7)	0.2 (0.1)	0.3 (0.1)	11.5 a (1.6)	12.1 (1.7)	0.2 (0.01)	0.1 (0.01)	8.5 bc (0.8)	8.8 (0.8)
40–60 cm	0.1 (0.04)	0.1 (0.01)	6.0 (0.3)	6.2 (0.3)	0.1 (0.04)	0.1 (0.01)	6.9 (0.2)	7.2 (0.3)	0.1 (0.02)	0.1 (0.01)	6.7 (0.3)	6.8 (0.3)	0.1 (0.01)	0.1 (0.01)	6.4 (0.5)	6.6 (0.5)
60–100 cm	0.1 (0.01)	0.1 (0.01)	4.8 (0.1)	4.9 (0.1)	0.1 (0.02)	0.1 (0.02)	5.1 (0.3)	5.3 (0.4)	0.1 (0.01)	0.1 (0.01)	4.9 (0.1)	5.0 (0.1)	0.1 (0.01)	0.04 (0.01)	5.7 (0.5)	5.8 (0.5)

Fallow-F: 4–7 years forest, Young-F: 20–30 years forest, Old-F: >50 years forest.

M-POM: 2000–250 μm, m-POM: 53–250 μm, Clay + silt: <53 μm

Different letters show significant differences for the vegetation of each soil depth, according to ANOVA and Tukey test (*P* < 0.05).

Values in parentheses indicate the standard errors (*N* = 4).

Visual inspection showed that most coarse or fine char materials in M-POM and m-POM samples were only in Cropland soils at 0–10 cm depth and Fallow-F soils at 0–5 cm depth. In contrast, most debris of M-POM and m-POM in Young-F and Old-F seemed to be fresh plant debris and not to be black in colour. For the upper layer (0–10 cm), total SOC stock was clearly larger in Fallow-F (33.4 ± 2.5 Mg C ha^−1^) and Cropland soils (31.2 ± 3.0 Mg C ha^−1^) than in Old-F (21.0 ± 3.6 Mg C ha^−1^), and Young-F (24.6 ± 2.7 Mg C ha^−1^) soils (Table 2). For the soil profile (100 cm depth), total SOC stock decreased in the order Fallow-F (136.6 ± 8.8 Mg C ha^−1^) > Cropland (122.2 ± 8.3 Mg C ha^−1^) and Young-F (119.1 ± 10.6 Mg C ha^−1^) > Old-F (106.4 ± 12.9 Mg C ha^−1^). This order was the same for SOC stock at 0–40 cm soil depth (Table 2). The total SOC stock in Fallow-F was the largest for most soil layers, except for the 0–10 cm layer in Cropland and the 20–40 cm layer in Young-F. The total SOC stock in Old-F was the smallest for all soil layers throughout the soil profile.

**Table 2:** Variations of soil organic carbon (SOC) derived from tropical forests (C3) and grassland (C4) in the whole soil profile under cropland and different fallow period sites in eastern Cameroon.

(Mg C ha-1)	Cropland			Fallow-F (4–7 y)			Young-F (20–30 y)			Old-F (>50 y)		
	Total	From C3	From C4	Total	From C3	From C4	Total	From C3	From C4	Total	From C3	From C4
0–10 cm	31.2 a (3.0)	25.2 a (1.7)	5.9 ab (0.7)	33.4 a (2.5)	25.2 a (2.0)	8.3 a (0.8)	24.6 ab (2.7)	16.4 b (2.4)	8.2 a (1.3)	21.0 b (3.6)	17.2 b (2.1)	3.8 b (0.7)
10–20 cm	19.4 ab (2.1)	11.8 ab (2.6)	7.6 ab (1.5)	24.5 a (1.8)	12.9 a (2.3)	11.6 a (1.3)	19.2 ab (2.0)	8.1 b (2.6)	11.0 a (1.7)	16.2 b (3.0)	10.5 ab (2.4)	5.7 b (1.6)
20–40 cm	25.5 ab (1.3)	15.1 (2.0)	10.4 b (1.6)	29.0 a (1.8)	13.4 (2.2)	15.6 a (1.2)	30.4 a (4.1)	13.7 (5.1)	16.7 a (3.1)	22.9 b (2.3)	13.7 (2.9)	9.1 b (1.9)
0–40 cm	76.1 ab (6.4)	52.1 a (3.8)	23.9 ab (2.3)	86.9 a (6.2)	51.4 a (3.8)	35.5 a (2.1)	74.2 ab (8.6)	38.2 b (6.1)	36.0 a (3.7)	60.0 b (8.8)	41.4 b (4.8)	18.6 b (2.6)
0–100 cm	122.2 ab (8.3)	82.0 a (5.2)	42.2 b (3.5)	136.6 a (8.8)	79.4 a (5.5)	57.2 a (3.8)	119.1 ab (10.6)	58.7 b (7.2)	60.4 a (5.8)	106.4 b (12.9)	71.4 ab (7.8)	35.0 b (4.1)

Fallow-F: 4–7 years forest, Young-F: 20–30 years forest, Old-F: >50 years forest.

From C3 and C4 indicate the amounts of SOC derived from C3 and C4 vegetation, respectively, according to ^13^C estimation.

Different letters show significant differences for the vegetation of each soil depth, according to ANOVA and Tukey test (*P* < 0.05).

Values in parentheses indicate the standard errors (*N* = 4).

### Natural abundance of ^13^C values in each fraction

**Figure 2:**
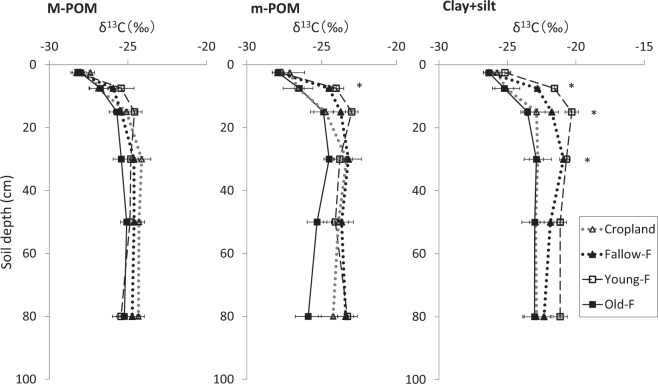
Fluctuation of δ13C (‰) values of soil organic carbon (SOC) in M-POM (250–2000 μm), m-POM (53–250 μm), and Clay + silt (<53 μm) fractions under cropland and different fallow period sites through the soil profile in eastern Cameroon. Bars indicate the standard error (*N* = 4). *Indicates the significant differences for the land management of each soil depth, according to ANOVA (*P* < 0.05).

Figure 2 presents the variations of natural abundance of ^13^C values of SOC in each fraction through the soil profile under different fallow stages. The ^13^C values in M-POM showed no clear difference between the sites, and these were within −27.4‰ to −28.3‰ in the surface layer (0–5 cm) and increased with soil depth to −24.3‰ to −25.4‰ in the deeper layer (60–100 cm depth). In the case of m-POM, ^13^C values were clearly larger in Fallow-F (−24.5‰ ± 0.8‰) and Young-F (−24.0‰ ± 0.5‰) than in Cropland (−26.6‰ ± 0.3‰) and Old-F (−26.5‰ ± 0.5‰) only at 5–10 cm depth, while there was no clear difference for the sites in the other soil layers.

The ^13^C values in Clay + silt were clearly different between the sites at 5–40 cm soil depth, while there was no clear difference in the surface layer (0–5 cm depth) and deeper layers (40–100 cm depth). The ^13^C values in Clay + silt decreased in the order Young-F > Fallow-F > Cropland and Old-F at 5–40 cm depth (*P* < 0.05). For example, ^13^C values in Clay + silt at 5–10, 10–20, and 20–40 cm depth of Young-F were −21.6‰ ± 0.2‰, −20.3‰ ± 0.4‰, and −20.7‰ ± 0.2‰, respectively, whereas they were −25.2‰ ± 0.6‰, −23.5‰ ± 0.2‰, and −22.9‰ ± 0.7‰ for Old-F, respectively. In addition, ^13^C values in Old-F and Cropland similarly fluctuated thorough the soil profile.

### Estimated SOC stock derived from C3 and C4 plants

**Figure 3:**
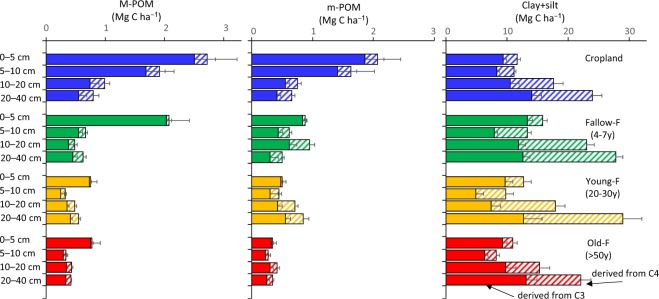
Distribution of soil organic carbon (SOC) derived from tropical forests (C3: filled bar) and grassland (C4: slash bar) in M-POM (250–2000 μm), m-POM (53–250 μm), and Clay + silt (<53 μm) fractions under cropland and different fallow period sites at each soil depth in eastern Cameroon.

Figure 3 presents the distribution of SOC derived from C3 and C4 plant in each fraction at 0–40 cm soil depth, and Table 2 shows the calculated SOC stock derived from C3 and C4 plants for each specific soil layer. In M-POM, most SOC was derived from C3 plants (92–98% at 0–5 cm depth and 68.5–77.8% at 20–40 cm depth). SOC derived from C3 plants in M-POM at 0–10 cm depth was clearly larger in Cropland (4.2 ± 0.9 Mg C ha^−1^) than in Young-F (1.0 ± 0.1 Mg C ha^−1^) and Old-F (1.0 ± 0.2 Mg C ha^−1^). In m-POM, the ratio of SOC derived from C3 plants decreased in Fallow-F and Young-F at 10–40 cm depths (e.g. 60.3–66.1%), whereas it was clearly high in Old-F (71.2–85.5%), and intermediate in Cropland (62.8–72.9%). SOC derived from C4 plants in m-POM at 10–40 cm was clearly larger in Young-F (0.6 ± 0.1 Mg C ha^−1^) than in Old-F (0.2 ± 0.1 Mg C ha^−1^). In Clay + silt, there was a larger contribution of C4-derived SOC especially in sub-surface layers (5–40 cm), whereas there was no clear effect in the surface layer (0–5 cm depth). The ratio of C4-derived SOC to the total SOC in Clay + silt at 5–40 cm depth was 35.4%, 48.0%, 55.0%, and 33.2% in Cropland, Fallow-F, Young-F, and Old-F, respectively. Within the upper layer (0–40 cm depth), SOC stock derived from C4 plants in Clay + silt was larger in Young-F (34.9 ± 3.3 Mg C ha^−1^) and Fallow-F (34.3 ± 1.9 Mg C ha^−1^) than in Old-F (18.1 ± 2.3 Mg C ha^−1^), while SOC stock derived from C3 plants in Clay + silt was larger in Fallow-F (45.8 ± 3.2 Mg C ha^−1^) and Cropland (42.4 ± 3.1 Mg C ha^−1^) than in Young-F (34.7 ± 4.2 Mg C ha^−1^).

Within the soil profile (100 cm depth: Table 2), SOC stock derived from C3 plants was clearly larger in Cropland (82.0 ± 5.2 Mg C ha^−1^) and Fallow-F (79.4 ± 5.5 Mg C ha^−1^) than in Young-F (58.7 ± 7.2 Mg C ha^−1^). In contrast, SOC stock derived from C4 plants was clearly larger in Young-F (60.4 ± 5.8 Mg C ha^−1^) and Fallow-F (57.2 ± 3.8 Mg C ha^−1^) than in Cropland (42.2 ± 3.5 Mg C ha^−1^) and Old-F (35.0 ± 4.1 Mg C ha^−1^).

## Discussion

Total SOC stock at both 100 cm depth and 0–40 cm depth decreased in the order Fallow-F > Cropland and Young-F > Old-F in the present study. This indicates that SOC stock peaked in the Fallow-F (i.e., the first stage of fallow succession), and then started to decrease with forest growth and maturity (i.e., middle to final stage of fallow succession) in this area. However, it should be noted that these results were obtained from the comparison of different fallow period sites, rather than from long-term, fixed-point observation. On the basis of the contribution of different C sources in Table 2, both C4- and C3-derived SOC was largest in the Fallow-F soil compared with other sites, indicating the significant contribution of both C4- and C3-derived SOC to the high SOC in Fallow-F. Because C4-derived SOC clearly increased from Cropland to Fallow-F, C4 grass invasion as a forest understory in Fallow-F is likely to have contributed to the substantial increase of SOC at this early fallow stage. In general, under the humid tropical conditions, the amount of OM incorporated into the soil annually is larger under the grass vegetation rather than under forest vegetation^14^. This is because forest fixes the C into the aboveground biomass especially in the growing and intermediate stages, such as Fallow-F and Young-F, whereas most C fixed by annual grass, including its root biomass, is incorporated into the soil every year^3,29^. Tonucci *et al*.^28^ also found a significant contribution of C4 pasture grass to SOC stock in agroforestry systems in Brazilian Cerrado. In addition, high N availability of Fallow-F might also stimulate the growth of C4 underforestry, resulting in larger C4 derived SOC^30^. Therefore, in the present study, invasion of C4 grass as forest understories is likely to have contributed to the increment of SOC stock, especially in the early fallow succession stage, with the largest SOC stock observed in Fallow-F.

In addition, added OM by slash-burn management (mostly C3-derived SOC) is likely to also contribute to the largest SOC stock in Fallow-F. This is because, considering the similar ^13^C values between the Cropland and Old-F in whole fractions through the soil profile (Fig. 2), the C source of SOC was not critically changed from Old-F to Cropland, i.e., the main C source is C3 plants. This indicates that the difference of SOC stock between the Cropland and Old-F must be mainly caused by applied OM after slash-burn management, such as burned forest biomass. This is consistent with the finding of larger C3-derived SOC in M-POM (mostly char material) in Cropland than that of Old-F. In addition, we also found that C3-derived SOC in each fraction of Cropland for the 0–40 cm depth was roughly similar to that of Fallow-F, whereas C3-derived SOC in M-POM and Clay + silt fractions for this depth clearly decreased from Fallow-F to Young-F (Fig. 3 and Table 2). This indicates that added OM by slash-burn management, including char, remained until the Fallow-F stage, though it began to be pulverized and/or lost in the Young-F stage. Zimmermann *et al*.^31^ estimated the average turnover time of purified wood C to be 67 years in a typical tropical savanna in Australia. Because of the more humid conditions in the present study, the coarse char material should be pulverized during a much shorter period, such as 67 years. Dong *et al*.^32^ also observed that biochar C loss was ca. 40% after 5 years in dry temperate cropland in China. Because we also found the decrease of C3-derived SOC in the M-POM and m-POM in the upper layer (0–10 cm depth) of Young-F and Old-F compared with those of Cropland, coarse char materials are likely to reduce in size and become part of the Clay + silt fraction during this period (i.e., from Cropland to Young-F)^33^. Lopez-Martin *et al*.^34^ observed the rapid decrease of coarse pyrogenic OM 7 years after forest fire event in Southern Spain, and indicated that the lack of mineral-pyrogenic OM association may have allowed rapid degradation of coarse pyrogenic OM or loss due to transport. On the basis of these results, in the present study, applied burned biomass (including char) should mostly remain in soil ca. 10–20 years after slash-burn management (such as the Cropland and Fallow-F stages in this study) under humid tropical climate conditions, possibly because of its purified structure (char) by fire^35–37^. Thus, in this study, added OM from slash-burn management was considered to still remain in Fallow-F, and it is likely to also contribute to the largest SOC stock in Fallow-F.

From Fallow-F to Young-F succession, we found that the C3-derived SOC clearly decreased, whereas C4-derived SOC remained stable (Table 2). This suggests the mechanism of decomposition of C3-derived SOC as explained above, and the existence of substantial C4 grass as a forest understory even in Young-F. In addition, we found that C4-derived SOC abruptly decreased from Young-F to Old-F succession, especially in Clay + silt (from 34.9 to 18.1 Mg C ha^−1^ at 0–40 cm depth, *P* < 0.05), whereas C3-derived SOC increased in Clay + silt (from 34.7 to 38.6 Mg C ha^−1^ at 0–40 cm depth). This indicates the disappearance of C4 grass understory in Old-F, possibly due to the crown closure and little insolation^38,39^.

Based on our field observations, there were few forest understories containing mainly C3 plants in Old-F, compared with that in Fallow-F and Young-F, for which the forest understory contained a higher amount of C4 grass. Thus, forest understories must strongly control the SOC stock in the middle to final stage of fallow succession in African tropical forest, and SOC stock was largest in the early fallow stage such as the 4–7-year fallow period, because of the added OM both of char by slash-burn management (C3-derived SOC) and of grass litter in forest understories (C4-derived SOC). Conversely, SOC stock was smallest in the final fallow stage (little C4 grass understories) such as after 50 years of fallow, i.e., in mature forest^40,41^. However, considering the amount of aboveground biomass C in each site, total C in Old-F (106.4 Mg C ha^−1^ in soil and 276.7 Mg C ha^−1^ in aboveground biomass) should be substantially larger than that in fallow-F (136.6 Mg C ha^−1^ in soil and 104.4 Mg C ha^−1^ in aboveground biomass). Therefore, to achieve the C sequestration to mitigate the climate change at ecosystem level (SOC + aboveground-C + belowground-C + deadwood-C + litter-C), a longer fallow period is likely to contribute to C sequestration in the tropical forest ecosystem^42–44^. Silatsa *et al*.^13^ also observed the significant contribution of aboveground biomass (cacao tree) to the C storage of agroforestry ecosystems in southern Cameroon.

There was a small amount of SOC present as M-POM and m-POM in Old-F and Young-F even in the surface layer, whereas SOC as M-POM and m-POM in Cropland was clearly larger and was mostly composed of C3 charred biomass rather than fresh plant debris (based on our visual observation). These results may indicate the rapid decomposition of normal plant debris and therefore applied OM, whichever from C4 or C3 plants, should be rapidly mineralised and moved into the Clay + silt fraction^45,46^. Yonekura *et al*.^27^ suggested the rapid mineralisation of applied litter under the humid tropical conditions, resulting in difficulties of C sequestration. In addition, because coarse char material in the M-POM and m-POM fractions in Cropland and Fallow-F was reduced in size and may move into the Clay + silt fraction in Young-F and Old-F, the Clay + silt-size char material is likely to have an influence on SOC stock and its stability for decomposition in Young-F and Old-F by association with mineral and/or OM. Thus, further study is necessary to assess the relationship between the particle char material and SOC stability in the Clay + silt fraction^24,47^.

## Conclusion

We evaluated the dynamics and the contributions of C4 grass and C3 forest to SOC stock during the fallow succession in eastern Cameroon by measuring SOC at sites with different fallow periods. It was found that the SOC stock was largest in Fallow-F. The finding that C4-derived C was the main contributor to restoration of the SOC in Fallow-F and Young-F sites (i.e., a 4–30 year fallow period) indicated that forest understories, particularly C4 grass, were important to restore the SOC stock during the early fallow period in African tropical forest of eastern Cameroon. In addition, OM added by slash-burn management still remained and contributed to larger SOC stock in Fallow-F. Based on these results, we put forward the hypothesis that a shorter fallow period, such as Fallow-F, might be sufficient to restore the soil fertility (or SOC restoration) for sustainable slash-burn agriculture in this area. Moreover, we also found that a longer fallow period is likely to contribute to C sequestration and climate change mitigation, because of the enhanced aboveground biomass C of mature forest. Further study is necessary to assess the above hypothesis, reveal the optimal fallow period for sustainable slash-burn agriculture in this area, and determine whether a shorter fallow period (such as Fallow-F in this study) in the tropical forest of eastern Cameroon can supply sufficient nutrients (such as N, P, and other minerals) to subsequent crop cultivation, including determination of an optimal cultivation period.

## Methods

### Site description and soil sampling

The study area was located in the Diang district of Bertour region (4°34ʹ56″N, 13°16ʹ00″E) in the eastern part of Cameroon, which contains the dominant geographic feature of the Cameroon Plateau. Oxisols are generally distributed throughout this region^48,49^, which stands at an elevation of approximately 650–700 m. The climate of the region is tropical monsoon with a mean annual temperature of 23.5 °C, and annual rainfall of 1350–1550 mm based on our former field survey^50^. This area usually experiences two rainy seasons, a short season from September to November and a long season from March to July.

In 2011, we collected soil samples from cropland and different fallow stage sites as follows: (1) cropland (Cropland), (2) short fallow forest (4–7 years; Fallow-F), (3) young forest (20–30 years; Young-F), and (4) old and matured forest (>50 years; Old-F), where the young and old forest developed after slash-burn cultivation. The detailed soil characteristics of Young-F and Old-F have previously been explained in Sugihara *et al*.^51^. In the Cropland site, local farmers generally cultivate cassava, banana, and groundnuts for 3–5 years, after opening and burning the mature forest such as Old-F. According to the interview with the local farmers, we selected the Cropland sites that had been in cultivation for 2–4 years, and sites that were mature forest (Old-F). In this region, to create the cultivation area, local farmer slashes only small- or middle-sized trees, but not the large trees (because of its difficulty by hand), and burns the material, and thus big trees still remain in the Cropland site even after slash-burn cultivation^12^. The Fallow-F, Young-F, and Old-F sites were divided by the period of fallow, which were representative of each natural fallow succession stages in this region, based on an interview with the local farmers. Our vegetation surveys (30 m × 30 m for each site, four replications) revealed no clear differences in the forest tree species composition between Young-F and Old-F, and the dominant vegetation was *Albizia zygia*, which predominates in the tree layer and *Myrianthus arboreus*. Estimated aboveground biomass in Young-F and Old-F were 221.1 Mg ha^−1^ (standard error, 84.0) and 276.7 Mg ha^−1^ (standard error, 44.7), respectively. In case of Fallow-F, the forest tree species composition was different from that of Young-F and Old-F, mainly comprising *Musanga cecropioides*, *Ficus exasperata*, and *Macaranga spinosa*, which are the typical pioneer species in early forest succession in this region. The estimated aboveground biomass in Fallow-F was 104.4 Mg ha^−1^ (standard error, 27.5). In addition, in the Fallow-F and Young-F sites, there were also C4 grass forest understories, such as *P. setaceum* and *I. cylindrica*, though there were few C4 grass forest understories in Old-F, possibly due to the crown closure and low-light conditions.

We made four pits (four replications) for each site (i.e., a total of 16 pits), and collected soil samples from each soil layer (0–5, 5–10, 10–20, 20–40, 40–60, and 60–100 cm). All sampling sites, except for one pit in Old-F, were within 10 km of each other (Supplemental Fig. 1). Soil samples were air dried, sieved to 2.0 mm, and then held at room temperature until used. All visible plant material larger than 2.0 mm sieve size was removed manually. We also collected two 100 cc soil cores at each soil depth for determination of the soil bulk density, to calculate the soil C stock in the soil profiles. Each of the experimental sites was relatively flat and had a flat area covering more than 1 km^2^. None of the pits contained gravel, and the clayey soils were classified as Typic Kandiudoxes^48^. Table S1 provides details of the physicochemical properties of soils under each site. The soil pH (H_2_O) was measured with a glass electrode (pH/ion meter 225, Iwaki Glass, Japan) at a soil:solution ratio of 1:5 (w:v) after shaking for 1 h. Particle size distribution was evaluated by a pipette method after removing OM with 5% H_2_O_2_ at 80 °C. To determine the cation-exchange capacity (CEC), we washed the residual soil with ethanol after ammonium acetate extraction and then extracted the remaining NH_4_^+^ with 10% NaCl, and determined the NH_4_^+^ concentration. Most physicochemical properties were not significantly different between the sites; however, clay contents of surface layers (0–5 and 5–10 cm depth) were smaller in Cropland than in Old-F and Young-F.

### Soil fractionation

The soil samples were manually fractionated by particle size, according to a procedure from Cambardella & Elliot^52^, as described by Sugihara *et al*.^51^. Briefly, 50 g of each soil sample was placed into a 250-mL plastic container and mixed with 150 mL of 0.05 mol L^−1^ NaCl solution. The soil suspension was then shaken for 16 h in a reciprocal shaker at 150 rpm, after which it was passed through a 0.25-mm sieve and rinsed with distilled water until a clear solution was obtained. Next, the sieved material was continuously passed through a 0.053-mm sieve and rinsed with distilled water until a clear solution was obtained. The material retained on each sieve was dried at 60 °C for 24 h as macro-POM (M-POM; 0.25–2.0 mm) and micro-POM (m-POM; 0.053–0.25 mm), containing the soil’s macro- and micro-particles, respectively; both also contained some plant debris. The soil slurry that passed through the 0.053-mm sieve was dried at 60 °C for 48 h to represent the clay + silt fraction (Clay + silt; <0.053 mm), which contained the mineral-associated OM. Generally, the M-POM fraction is defined as easily mineralisable OM, and the Clay + silt fraction includes residual or stable OM^53^. In the present study, M-POM mainly contained plant debris and its char, whereas m-POM mainly contained small particles of ash. The recovery ratio for this procedure was 93–99% of total mass and 93–101% of total carbon (TC).

### Soil analysis

All the fractionated samples were completely ground to powder using a tungsten mortar and pestle. The TC content for whole soil and for fractionated soil samples were determined with a CN analyser (Vario Max CN, Elementar, Hanau, Germany).

For stable C isotope determinations, oven-dried fractionated samples were analysed for δ^13^C values using a stable isotope ratio mass spectrometer (IsoPrime100, Elementar UK Ltd, Cheadle, UK) operating in continuous flow mode, coupled with a CN analyser (Vario Max CN, Elementar, Hanau, Germany). Carbon isotope ratios are presented in δ-notation:

1 $${{\rm{\delta }}}^{13}{\rm{C}}=([{{\rm{R}}}_{{\rm{sample}}}-{{\rm{R}}}_{{\rm{std}}}]/{{\rm{R}}}_{{\rm{std}}})\times {10}^{3}$$

where R_sample_ is the ^13^C/^12^C ratio of the sample, and R_std_ is the ^13^C/^12^C ratio of the international standard PDB^54^. SOC content derived from the C4 grass plants (*Pennisetum* spp., and *Imperata* spp.) or from the C3 forest plants (*Albizia* spp., and *Myrianthus* spp.) was estimated based on the equations^18,20,55^:

2 $${\rm{C}}4 \mbox{-} {\rm{derived}}\,{\rm{SOC}}\,({\rm{g}}\,{{\rm{kg}}}^{-1})=({\rm{\delta }}-{{\rm{\delta }}}_{{\rm{T}}})/({{\rm{\delta }}}_{{\rm{G}}}-{{\rm{\delta }}}_{{\rm{T}}})\times {\rm{total}}\,{\rm{C}}\,({\rm{g}}\,{{\rm{kg}}}^{-1})$$

3 $${\rm{C}}3 \mbox{-} {\rm{derived}}\,{\rm{SOC}}\,({\rm{g}}\,{{\rm{kg}}}^{-1})={\rm{total}}\,{\rm{C}}-{\rm{C}}4 \mbox{-} {\rm{derived}}\,{\rm{SOC}}$$

where δ is the δ^13^C of a given samples, δ_T_ is a composite sample of the C3 forest plants (−28.5‰) and δ_G_ is a composite sample of C4 grass (−14.6‰). Both δ_T_ and δ_G_ were average (*N* = 5) of plant litter for C4 and C3 vegetation, and both values were consistent with the previous study in eastern Cameroon^22^ and therefore, in this study, we assumed that the added OM of C3 and C4 plants had the above δ^13^C value, respectively.

### Statistical analysis

All statistical analyses were performed with SYSTAT 13.5 (SYSTAT Software, Richmond, CA, USA). All data are expressed on a dry-weight basis. To compare the δ13C values obtained in each fraction, we conducted a one-way analysis of variance (ANOVA) to assess the significance of differences between the experimental sites. When ANOVA indicated significant differences, mean comparisons were performed with the Tukey multiple comparison test. We also conducted the same multiple analysis to the total SOC, and calculated C derived from C3 and C4 between the experimental sites. In all cases, *P* < 0.05 was considered significant.

## Supplementary information

Supplemental information
