# Supplementary material for: Forest understories controlled the soil organic carbon stock during the fallow period in African tropical forest: a 13C analysis
Source: Sci Rep. 2019 Jul 8;9:9835. doi: 10.1038/s41598-019-46406-2 (PMC6614393; doi:10.1038/s41598-019-46406-2)

Forest understories controlled the soil organic carbon stock during the fallow period in African tropical forest: a ^{13}C analysis

Soh Sugihara[1*], Makoto Shibata[2,3], Antoine D. Mvondo Ze[4], Haruo Tanaka[1], Takashi Kosaki[5], and Shinya Funakawa[2]

[1] Graduate School, Institute of Agriculture, Tokyo University of Agriculture and Technology, 183-8509, Japan
[2] Graduate School of Global Environmental Studies, Kyoto University, 606-8501, Japan
[3] Department of Agro-Food Science, Niigata Agro-Food University, 950-3102, Japan
[4] Faculté d' Agronomie, Université de Dschang, B.P. 67, Dschang, Cameroon
[5] Graduate School of Global Liberal Arts, Aichi University, 453-8777, Japan

[*]Corresponding author: Dr. Soh SUGIHARA
Graduate School, Institute of Agriculture, Tokyo University of Agriculture and Technology, Saiwaicho 3-5-8, Fuchu, Tokyo, 183-8509, Japan
Telephone: +81-42-367-5676 Fax: +81-42-367-5676 E-mail: sohs@cc.tuat.ac.jp

Supplemental Table 1. Soil physicochemical properties under different land management through the profiles in eastern Cameroon.

	Soil pH (H_2O)				Clay (%)				CEC ($cmol_c\ kg^{-1}$)			
	Cropland	Fallow-F	Young-F	Old-F	Cropland	Fallow-F	Young-F	Old-F	Cropland	Fallow-F	Young-F	Old-F
0-5 cm	5.5 (0.3)	5.7 (0.4)	5.3 (0.2)	4.8 (0.2)	43.7 (3.9) b	51.9 (1.8) ab	53.6 (0.9) a	57.9 (1.4) a	11.1 (1.6)	16.8 (1.3)	13.5 (2.6)	10.9 (1.9)
5-10 cm	5.4 (0.3)	5.5 (0.3)	5.2 (0.1)	4.6 (0.2)	44.5 (3.9) b	52.6 (1.7) ab	56.2 (1.1) a	58.7 (1.2) a	9.3 (0.5)	11.3 (1.1)	8.5 (0.9)	9.1 (2.6)
10-20cm	5.2 (0.3)	5.3 (0.2)	5.1 (0.1)	4.6 (0.2)	50.0 (4.5)	56.8 (1.9)	57.0 (0.8)	58.3 (1.3)	7.2 (0.6)	10.0 (1.2)	7.6 (0.4)	8.4 (1.7)
20-40cm	5.0 (0.2)	5.2 (0.2)	5.2 (0.1) c	4.7 (0.1)	63.3 (3.9)	65.8 (3.0)	65.1 (3.0)	68.4 (0.8)	9.0 (0.8)	8.0 (0.6)	7.4 (0.5)	8.1 (0.8)
40-60cm	5.0 (0.1)	5.3 (0.2)	5.3 (0.1)	4.6 (0.1)	69.4 (1.7)	69.6 (2.8)	69.8 (3.1)	73.7 (1.1)	8.3 (0.6)	8.4 (0.7)	8.1 (1.1)	7.3 (1.0)
60-100cm	5.1 (0.2)	5.4 (0.2)	5.5 (0.1)	4.8 (0.1)	71.7 (1.3)	72.5 (2.2)	73.0 (3.1)	75.2 (1.3)	9.1 (1.0)	9.6 (1.4)	8.5 (0.7)	7.8 (1.2)

Fallow-F: 3~5 years forest, Young-F: 20–30 years forest, Old-F: >50 years forest.

Different letters show significant differences for the vegetation of each soil depth, according to ANOVA and Tukey test ($P < 0.05$).

Numbers shown in parenthese indicate the standard errors ($N = 4$).

Supplemental Table 2. Total C contents (TC), total N contents (TN), and C:N ratio of each fraction of soils under different land management through the profiles in eastern Cameroon.

TC ($gC\ kg^{-1}$ soill)	Cropland				Fallow-F (4-7y)				Young-F (20-30y)				Old-F (>50y)			
	M-POM	m-POM	Clay+silt	Total	M-POM	m-POM	Clay+silt	Total	M-POM	m-POM	Clay+silt	Total	M-POM	m-POM	Clay+silt	Total
0-5 cm	4.9 (1.6)a	3.7 (1.0) a	21.2 (1.3) b	29.9 (3.8) b	4.2 (0.7) a	3.6 (0.1) a	32.2 (1.5) a	40.1 (2.1) a	1.5 (0.2) b	1.9 (0.3) b	26.3 (2.6) ab	28.5 (3.3) b	1.5 (0.3) b	1.2 (0.3) b	21.8 (3.1) b	24.5 (3.3) b
5-10 cm	3.2 (1.3) a	2.7 (1.0) a	18.6 (1.7) ab	24.6 (3.7) a	1.1 (0.1) b	1.0 (0.1) b	22.5 (2.0) a	24.6 (2.0) a	0.6 (0.1) c	0.8 (0.2) b	18.0 (2.1) ab	19.4 (2.4) ab	0.5 (0.1) c	0.4 (0.1) c	13.9 (2.8) b	14.9 (2.9) b
10-20cm	0.7 (0.2)	0.5 (0.1)	12.9 (1.4) b	14.1 (1.6) ab	0.8 (0.1)	0.8 (0.2)	18.4 (1.0) a	19.9 (1.2) a	0.4 (0.1)	0.6 (0.1)	15.6 (1.6) ab	16.6 (1.7) ab	0.3 (0.01)	0.3 (0.1)	12.1 (2.2) b	12.8 (2.3) b
20-40cm	0.3 (0.1)	0.2 (0.03)	8.2 (0.3) c	8.7 (0.4)	0.2 (0.1)	0.2 (0.03)	10.1 (0.6) ab	10.5 (0.7)	0.2 (0.1)	0.3 (0.1)	11.5 (1.6) a	12.1 (1.7)	0.2 (0.01)	0.1 (0.01)	8.5 (0.8) bc	8.8 (0.8)
40-60cm	0.1 (0.04)	0.1 (0.01)	6.0 (0.3)	6.2 (0.3)	0.1 (0.04)	0.1 (0.01)	6.9 (0.2)	7.2 (0.3)	0.1 (0.02)	0.1 (0.01)	6.7 (0.3)	6.8 (0.3)	0.1 (0.01)	0.1 (0.01)	6.4 (0.5)	6.6 (0.5)
60-100cm	0.1 (0.01)	0.1 (0.01)	4.8 (0.1)	4.9 (0.1)	0.1 (0.02)	0.1 (0.02)	5.1 (0.3)	5.3 (0.4)	0.1 (0.01)	0.1 (0.01)	4.9 (0.1)	5.0 (0.1)	0.1 (0.01)	0.04 (0.01)	5.7 (0.5)	5.8 (0.5)

TN ($g\ N\ kg^{-1}$ soil)	Cropland				Fallow-F (4-7y)				Young-F (20-30y)				Old-F (>50y)			
	M-POM	m-POM	Clay+silt	Total	M-POM	m-POM	Clay+silt	Total	M-POM	m-POM	Clay+silt	Total	M-POM	m-POM	Clay+silt	Total
0-5 cm	0.3 (0.1) a	0.3 (0.1) a	1.9 (0.2) b	2.5 (0.3) b	0.3 (0.1) a	0.3 (0.03) a	2.9 (0.2) a	3.4 (0.2) a	0.1 (0.02) b	0.1 (0.02) b	2.3 (0.3) ab	2.4 (0.3) b	0.1 (0.02) b	0.1 (0.02) b	2.1 (0.3) b	2.2 (0.3) b
5-10 cm	0.2 (0.04)a	0.2 (0.03) a	1.7 (0.1)	2.1 (0.3)	0.1 (0.01) b	0.1 (0.02) b	1.8 (0.2)	1.9 (0.2)	0.03 (0.01) b	0.04 (0.01) b	1.5 (0.2)	1.5 (0.2)	0.03 (0.01) b	0.02 (0.01) b	1.2 (0.2)	1.3 (0.2)
10-20cm	0.03 (0.01)	0.03 (0.01)	1.1 (0.1) b	1.1 (0.1) b	0.03 (0.01)	0.01 (0.00)	1.5 (0.1) a	1.6 (0.1) a	0.02 (0.01)	0.03 (0.01)	1.2 (0.1) b	1.3 (0.1) b	0.02 (0.01)	0.02 (0.01)	1.1 (0.2) b	1.1 (0.2) b
20-40cm	0.01 (0.01)	0.01 (0.00)	0.7 (0.1)	0.8 (0.1)	0.01 (0.01)	0.01 (0.00)	0.9 (0.1)	0.9 (0.1)	0.01 (0.00)	0.02 (0.01)	0.9 (0.1)	1.0 (0.1)	0.01 (0.00)	0.01 (0.01)	0.8 (0.1)	0.8 (0.1)
40-60cm	0.01 (0.00)	0.01 (0.00)	0.6 (0.03)	0.6 (0.03)	0.01 (0.00)	0.01 (0.00)	0.7 (0.01)	0.7 (0.01)	0.01 (0.00)	0.01 (0.00)	0.6 (0.04)	0.6 (0.04)	0.01 (0.00)	0.01 (0.00)	0.6 (0.1)	0.6 (0.1)
60-100cm	0.01 (0.00)	0.01 (0.00)	0.4 (0.01)	0.4 (0.01)	0.01 (0.00)	0.01 (0.00)	0.5 (0.02)	0.5 (0.02)	0.01 (0.00)	0.01 (0.00)	0.5 (0.03)	0.5 (0.03)	0.01 (0.00)	0.01 (0.00)	0.5 (0.1)	0.5 (0.1)

C:N ratio	Cropland				Fallow-F (4-7y)				Young-F (20-30y)				Old-F (>50y)			
	M-POM	m-POM	Clay+silt	Total	M-POM	m-POM	Clay+silt	Total	M-POM	m-POM	Clay+silt	Total	M-POM	m-POM	Clay+silt	Total
0-5 cm	16.5 (1.3)	14.8 (1.4)	11.1 (0.3)	11.9 (0.3)	16.6 (1.0)	15.3 (1.0)	11.3 (0.4)	12.0 (0.5)	15.2 (0.6)	15.1 (0.6)	11.3 (0.3)	11.7 (0.2)	20.1 (2.4)	16.4 (1.9)	10.7 (0.5)	11.2 (0.5)
5-10 cm	17.1 (1.1)	15.6 (1.3)	11.2 (0.3) ab	11.8 (0.4)	22.6 (2.3)	21.5 (2.5)	12.4 (0.6) a	12.8 (0.7)	18.6 (1.4)	21.4 (2.1)	12.4 (0.3) a	12.7 (0.3)	18.3 (2.3)	18.9 (2.9)	10.9 (0.5) b	11.2 (0.5)
10-20cm	21.5 (2.1)	17.1 (1.3)	12.0 (0.7)	12.3 (0.7)	22.8 (1.5)	22.0 (2.6)	12.2 (0.2)	12.6 (0.3)	19.0 (1.2)	21.3 (1.5)	12.5 (0.3)	12.8 (0.3)	20.8 (3.7)	20.7 (3.3)	11.4 (0.7)	11.7 (0.7)
20-40cm	28.4 (5.5)	18.5 (1.5)	11.3 (0.4)	11.5 (0.4)	19.9 (2.5)	22.9 (2.2)	11.5 (0.3)	11.7 (0.3)	17.9 (1.5)	22.8 (1.1)	12.1 (0.5)	12.3 (0.5)	23.5 (5.4)	21.4 (4.2)	11.2 (0.6)	11.4 (0.6)
40-60cm	13.6 (1.1)	17.2 (1.0)	11.0 (0.3)	11.1 (0.3)	17.6 (3.9)	18.2 (2.6)	10.3 (0.2)	10.5 (0.2)	14.8 (1.3)	27.0 (4.4)	10.9 (0.3)	11.0 (0.3)	22.3 (6.2)	18.8 (5.5)	11.1 (0.7)	11.2 (0.7)
60-100cm	14.7 (1.9)	13.3 (1.7)	10.7 (0.4)	10.8 (0.4)	12.1 (2.8)	11.8 (1.6)	9.9 (0.3)	9.9 (0.4)	12.9 (2.4)	17.3 (1.5)	9.8 (0.2)	9.9 (0.1)	17.0 (3.2)	14.6 (5.0)	11.1 (0.7)	11.1 (0.6)

Fallow-F: 4~7 years forest, Young-F: 20–30 years forest, Old-F: >50 years forest.

M-POM: 2000–250μm, m-POM: 53–250μm, Clay+silt: <53μm

Different letters show significant differences for the vegetation of each soil depth, according to ANOVA and Tukey test ($P < 0.05$).

Values in parenthese indicate the standard errors ($N = 4$).

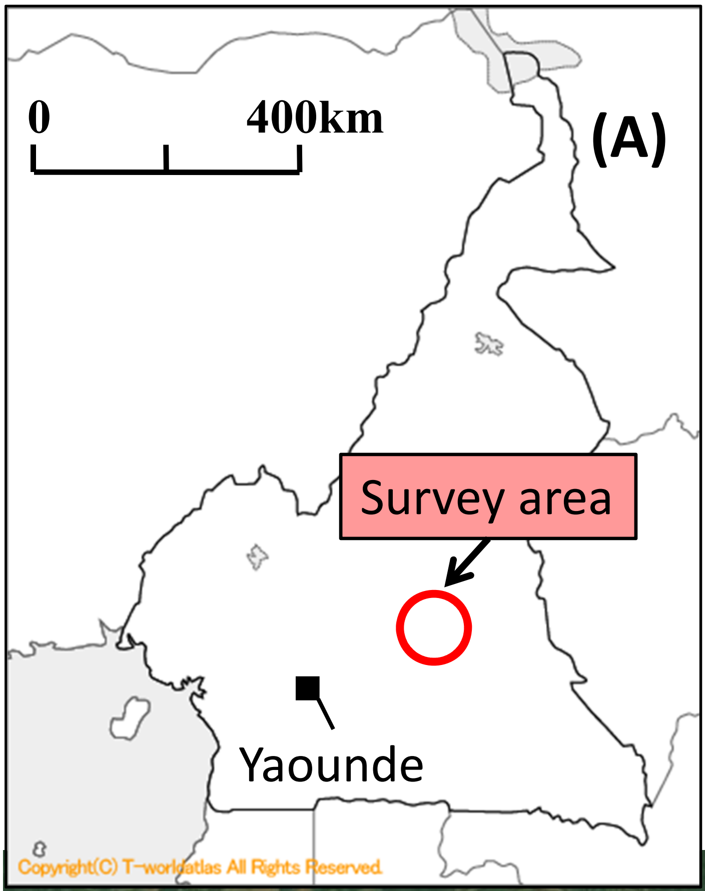

Supplemental Fig. 1
Map of survey area in Cameroon (A) and detailed position of soil profiles under different land management.
Cropland (White pin), Fallow-F (Red pin), Young-F (Yellow pin) and Old-F (Blue pin).
Supplemental Fig. 1A was made by using free map from the Website (http://www.sekaichizu.jp/).
Supplemental Fig. 1B was made with Google Earth Pro.
They allow all the users to use their map for any purposes including commercial use.

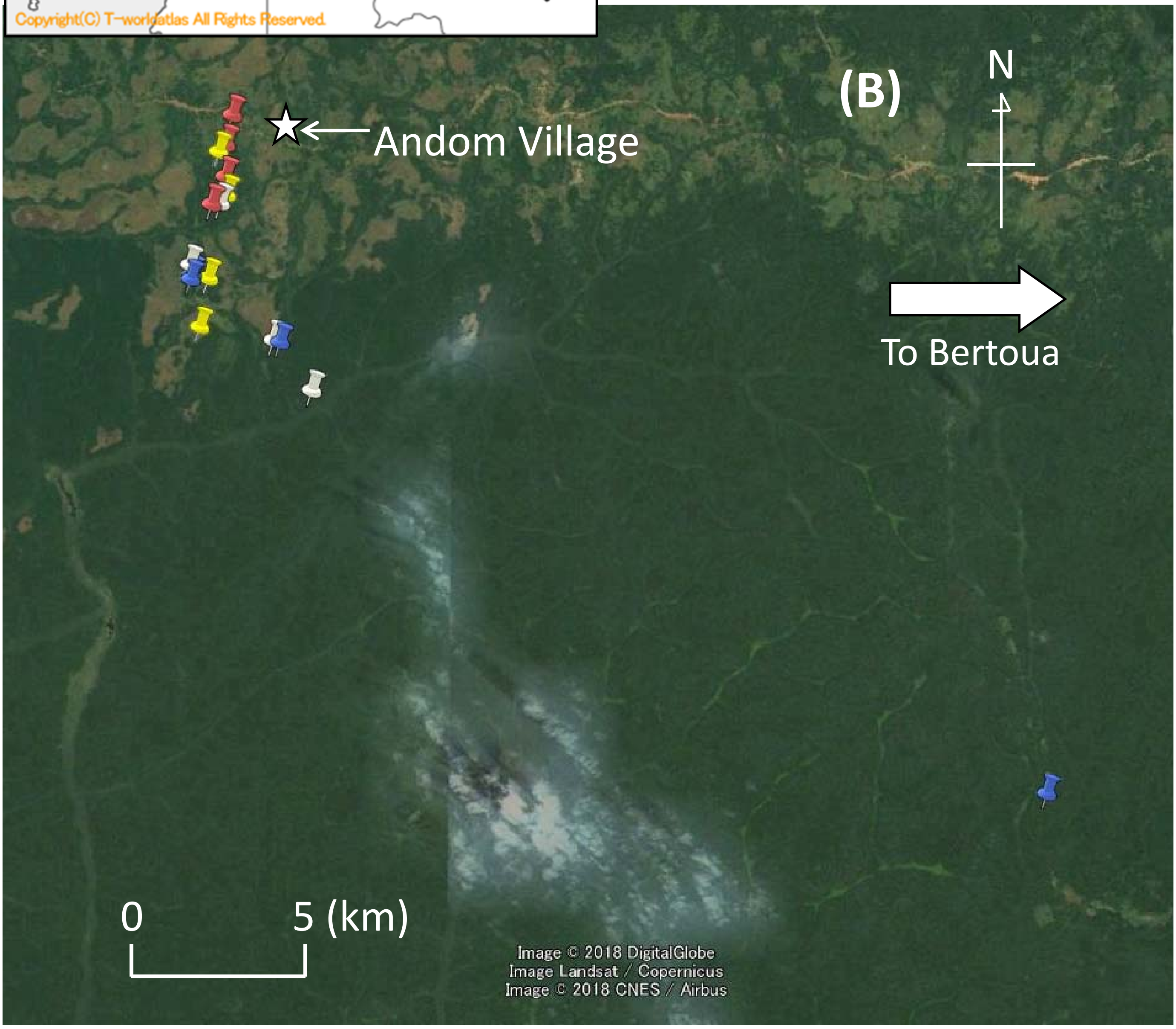

Supplement: Supplementary file 1 — Supplemental information [file 41598_2019_46406_MOESM1_ESM.pdf]
